# Population admixtures in medaka inferred by multiple arbitrary amplicon sequencing

**DOI:** 10.1038/s41598-022-24498-7

**Published:** 2022-11-21

**Authors:** Shingo Fujimoto, Hajime Yaguchi, Taijun Myosho, Hiroaki Aoyama, Yukuto Sato, Ryosuke Kimura

**Affiliations:** 1 Graduate School of Medicine, University of the Ryukyus, Nishihara, Okinawa 903-0125 Japan; 2 Present Address: Research Laboratory Center, Faculty of Medicine, University of the Ryukyus, Nishihara, Okinawa 903-0213 Japan; 3 Tropical Biosphere Research Center, University of the Ryukyus, Nishihara, Okinawa 903-0213 Japan; 4 Present Address: Department of Bioscience, School of Science and Technology, Kwansei Gakuin University, Nishihara, Hyogo 669-1330 Japan; 5 Laboratory of Molecular Reproductive Biology, Institute for Environmental Sciences, University of Shizuoka, Nishihara, 422-8526 Japan; 6 Center for Strategic and Research Center, University of the Ryukyus, Nishihara, Okinawa 903-0213 Japan; 7 Research Planning Office, University of the Ryukyus, Nishihara, Okinawa 903-0213 Japan

**Keywords:** Zoology, Ichthyology, Biological techniques, Experimental organisms, Ecology, Conservation biology, Molecular ecology

## Abstract

Cost-effective genotyping can be achieved by sequencing PCR amplicons. Short 3–10 base primers can arbitrarily amplify thousands of loci using only a few primers. To improve the sequencing efficiency of the multiple arbitrary amplicon sequencing (MAAS) approach, we designed new primers and examined their efficiency in sequencing and genotyping. To demonstrate the effectiveness of our method, we applied it to examining the population structure of the small freshwater fish, medaka (*Oryzias latipes*). We obtained 2987 informative SNVs with no missing genotype calls for 67 individuals from 15 wild populations and three artificial strains. The estimated phylogenic and population genetic structures of the wild populations were consistent with previous studies, corroborating the accuracy of our genotyping method. We also attempted to reconstruct the genetic backgrounds of a commercial orange mutant strain, Himedaka, which has caused a genetic disturbance in wild populations. Our admixture analysis focusing on Himedaka showed that at least two wild populations had genetically been contributed to the nuclear genome of this mutant strain. Our genotyping methods and results will be useful in quantitative assessments of genetic disturbance by this commercially available strain.

## Introduction

The development of high throughput sequencing techniques has facilitated genotyping by sequencing (GBS)^1,2^. Sequence outputs of short-read sequencers have increased dramatically, and the indexed library constructions for sample multiplexing have facilitated the sequencing for hundreds of samples at once^3^. As a result of these technological advances, population data of single nucleotide variations (SNVs) in the nuclear genome have been widely used to analyze population structure in a broad range of taxa^4^. Inference of past demographic events based on genome-wide SNVs is becoming fundamental to our understanding of the ecology and evolution of species in the wild.

To obtain genome-wide SNVs, whole-genome re-sequencing (WGS) is widely used in organisms, such as insects, fishes, and plants^5–8^. In studies of non-model organisms, researchers have also frequently used restriction site-associated DNA sequencing methods (RAD-seq), which rely on deeply sequencing only those genomic regions that are adjacent to restriction sites^4,9–11^. Because WGS and RAD-seq require a relatively large amount of high-quality genomic DNA (e.g., 100 ng per sample for RAD-seq and up to 1000 ng for WGS)^2,4,11,12^, these methods are sometimes difficult to be employed when the amount of DNA is limited.

PCR amplicon-based sequencing methods have been proposed as a complementary approach to WGS and RAD-seq. Some amplicon-based GBS methods rely on multiplex primer sets that focus on specific target sequences, but such methods require the reference genome to design the primers^13,14^. Alternatively, short primers which have a low priming specificity can arbitrarily amplify multiple regions in the target genome; for example, sequencing methods such as MIG-seq, RAMseq, and Gras-Di^15–17^. We referred this approach as multiple arbitrary amplicon sequencing (MAAS), after multiple arbitrary amplicon profiling (MAAP) that has been used previously to detect polymorphic DNA fragments by gel electrophoresis^18^. MAAS methods have advantages that the designed primer sequences can be universally used for many taxa and that they only require a simple library construction using two steps PCR^17^. In addition, PCR amplicon can be applied for cases where input DNA amounts are limited; for example, small organisms such as plankton, fish eggs, insects^19^, and tissues with low DNA content such as feces, hairs, and feathers^20,21^, or small portions of museum specimens.

Previous studies based on MAAS produced only 10^2^–10^3^ SNVs using relatively low throughput sequencers, such as the Roche 454 and Illumina MiSeq systems^15,17,22^. However, a larger number of SNVs are generally required to estimate past demographic events including population admixture. For example, admixture analyses using *Patterson’s D* and related *F* statistics^23–26^ usually require at least 10^3^–10^4^ SNVs (10^2^–10^3^ informative sites) to obtain a statistically reliable estimation. Therefore, in order to apply MAAS to population admixture analyses, technical challenges for improving genotyping efficiency are needed. In sequencing applications using the MAAS method, the number of SNVs may not increase linearly, even when more sequence-read data are generated. This is because of the presence of amplification bias among multiple PCR products, where only some products are deeply sequenced and these sequences then account for a large proportion of the sequence output. Another problem is that SNV sites containing no missing genotype calls dramatically decrease as the number of samples increases^17,22^. In this study, we designed primers with a low amplification bias to increase genotyping efficiency in MAAS, and then compared the genotyping efficiency of these primers with MIG-seq primers that had been developed previously^17^.

Establishing an experimental system based on the MAAS approach, the present study also performed population genetics analyses on medaka (*Oryzias latipes* species complex), a small freshwater fish for which a high-quality reference genome already exists^27^. Medaka, or *O. latipes* species complex are distributed in rice fields and shallow wetlands throughout Japan, Korea, and China^28^. Molecular phylogenetic analyses have revealed that Japanese populations of the *O. latipes* species complex consist of two genetically distinct groups^10,14,29,30^; a ‘Northern Japan group’ or *O. sakaizumii*^31^, which is distributed along the Sea of Japan coast, and a ‘Southern Japan group’ or *O. latipes*, which is distributed along the Pacific coast and the East China Sea coast throughout Japan.

Previously, population studies on lab-stock medaka strains derived from wild populations have been conducted using mitochondrial haplotypes and nuclear genome SNVs genotyped by the ddRAD-seq method^10,30^. However, the genetic diversity within a wild population and the genetic differentiation between wild populations cannot be estimated from the study using lab-stock strains. Therefore, we used individuals from wild medaka populations in Japan and investigated their phylogeny and population structure to reconstruct possible scenarios for the formation of the wild populations.

In addition, we investigate the genetic background of a commercial orange mutant strain, Himedaka. The strain has been widely cultured in Japan and kept as an ornamental fish since before the Edo era in the nineteenth century. Based on cytochrome *b* gene mitotypes, Himedaka has been classified as belonging to *O. latipes* (Table S11)^32,33^. Although the Himedaka strain has been widely used as an ornamental fish and an experimental model, the genetic background of the nuclear genome has not yet been investigated. Moreover, it has been reported that anthropogenic introductions of the Himedaka strain may have caused a genetic disturbance in wild populations^32–34^. If the genetic characteristics of the Himedaka strain can be clarified, then this information will be useful for detecting genetic disturbance by this strain in wild populations.

## Results and discussion

### Establishment of an efficient genotyping method based on the MAAS approach

**Figure 1:**
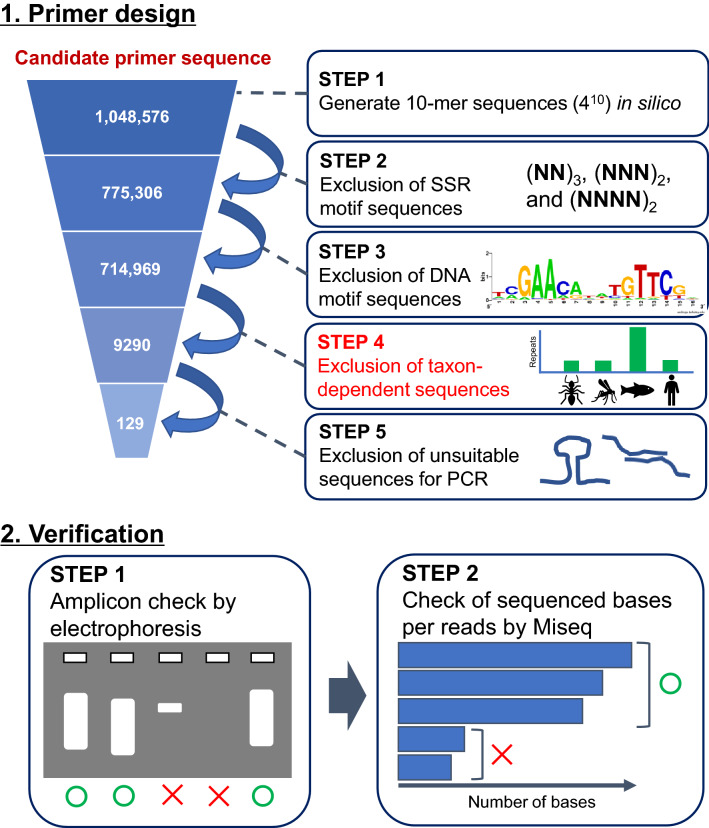
Workflow showing primer design and verification by multiple arbitrarily amplicon sequencing.

**Figure 2:**
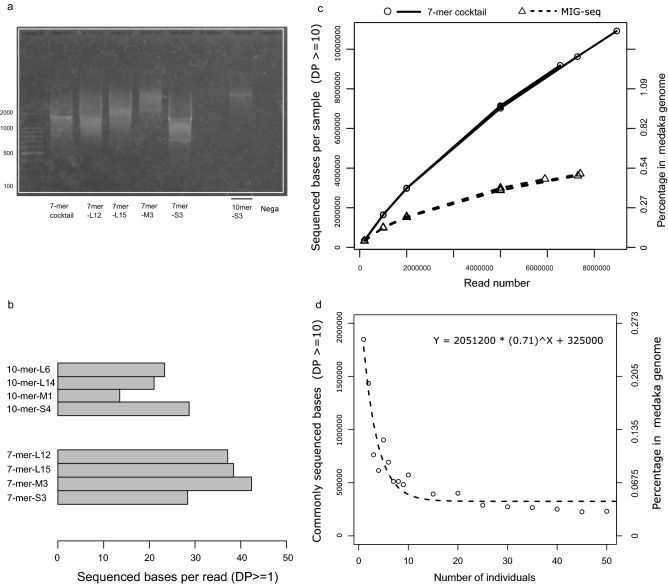
Sequencing performance of the developed primers. (**a**): Representative example of electrophoresis of each primer set (The white line represented cropped region to exclude the empty lanes, original uncropped gel image is presented in Supplementary information in Fig. S4); (**b**): The sequenced bases per read by Miseq output; (**c**): Relationship between read number and total sequenced bases obtained using 7-mer and MIG-seq cocktails. (**d**): Reduction curve of commonly sequenced bases among randomly selected individuals of *O. latipes*.

MAAS, based on short primers, can arbitrarily amplify multiple regions in the target genome^15–17^. To increase the genotyping efficiency, we attempted to design primers with a low amplification bias as follows (details in the Materials and Methods, Fig. 1): (1) All possible 10-mer sequence combinations were generated in silico, (2) The sequences containing simple sequence repeats were excluded, (3) sequences containing a functional motif of a transcription factor-binding site were excluded (Table S12), (4) taxon-dependent repeated sequences were excluded based on the *k*-mer frequency of reference genomes in 17 species from multiple phyla, and (5) self-complementary sequences were also excluded to avoid synthesizing primer dimers.. Twenty-four candidate sequences for 10-mer primers were designed, and their first seven bases were used as 7-mer primers (Fig. 1, Table S1). Then, we confirmed PCR amplification of these primers and selected eight sequences (four sequences each for 10-mer and 7-mer) that uniformly amplified 500–2000 bp products by electrophoresis on agarose gels (Figs. 2a, S4). We compared the sequencing performance of each primer set and found that the number of sequenced bases (depth ≥ 1) per read was higher for the 7-mer primers than for the 10-mer primers (Fig. 2b). Based on these findings, we used a primer cocktail consisting of four 7-mer primer sequences (Table 1).

**Table 1:** Primer sequences used for the 7-mer cocktail.

Name	Sequences (5′–3′) (adapter + spacer + target)
**Forward primers**	
7mer-S3-f	CGCTCTTCCGATCTNNNNNNNGTCGCCC
7mer-M3-f	CGCTCTTCCGATCTNNNNNNNTTTGATC
7mer-L12-f	CGCTCTTCCGATCTNNNNNNNTTTATGT
7mer-L15-f	CGCTCTTCCGATCTNNNNNNNTTTTTGT
**Reverse primers**	
7mer-S3-r	TGCTCTTCCGATCTNNNNNNNGTCGCCC
7mer-M3-r	TGCTCTTCCGATCTNNNNNNNTTTGATC
7mer-L12-r	TGCTCTTCCGATCTNNNNNNNTTTATGT
7mer-L15-r	TGCTCTTCCGATCTNNNNNNNTTTTTGT

One of previously proposed MAAS methods, MIG-seq, uses inter-simple sequence repeats (ISSRs) as primers (Table S3)^17^. To compare our 7-mer primer cocktails with the MIG-seq primer cocktails in the sequencing efficiency, we investigated the relationship between the read number and sufficiently sequenced bases (i.e., depth ≥ 10). In the mapping process, the proportion of uniquely mapped reads was higher for the MIG-seq cocktail (70%) than for the 7-mer cocktail (63%) (Table 2). However, when we considered sequences with depth ≥ 10 under a practical range of read numbers from 0.2 to 6.0 million reads, the 7-mer cocktail had coverage bases per read that were two to three times greater than those obtained using the MIG-seq cocktail (Fig. 2c).

**Table 2:** Results of sequence quality filtering and mapping on the reference genome.

Primer	Individual	Fastq	Quality check *1		Mapping *2			
		Raw reads (A)	Passed reads (B)	B/A	Properly paired	Uniquely mapped (C)	C/B	(C/A)
7-mer cocktail	Kun09	13,510,718	11,770,022	0.87	10,477,920	8,545,086	0.73	0.63
7-mer cocktail	Kun10	17,388,764	15,186,928	0.87	13,669,208	10,957,140	0.72	0.63
7-mer cocktail	Kun12	14,529,020	12,912,674	0.89	11,597,060	9,284,008	0.72	0.64
MIG-seq	Kun09	11,365,934	9,990,846	0.88	8,936,336	7,897,270	0.79	0.69
MIG-seq	Kun10	13,538,806	11,851,104	0.88	10,527,678	9,404,124	0.79	0.69
MIG-seq	Kun12	13,249,288	11,715,710	0.88	10,397,200	9,305,213	0.79	0.70

*1: Primer sequences, Illumina adapters, and low-quality reads were trimmed. The phred score less than 15 for more than 40% bases, or a read with fewer than 15 bases. *2: Mapping on the reference genome was conducted using BWA mem. A summary of the mapped reads was obtained using SAMtools with the flagstat command.

Our results also showed that the sequenced bases obtained using both the 7-mer and MIG-seq primer cocktails increased linearly to at least 6.0 million reads per sample without reaching saturation (Fig. 2c). This indicates a potential ability of MAAS methods. MAAS methods have been usually conducted using the Illumina MiSeq platform, in which the read number ranges from 10^4^ to 10^5^ per sample^17,22,35^. However, our result suggests that there is a room for increasing the number of the genotyped sites if the sequencing throughput increases.

The difference in the results between the 7-mer and MIG-seq primer cocktails was likely caused by amplification bias among the PCR products. Sequences with a suitable depth in the 7-mer cocktail (depth = 10 to 100) were nearly twice as large as those obtained using the MIG-seq cocktail, whereas sequences with extremely high depth (depth ≥ 500 or 1000) were more abundant when using the MIG-seq cocktail (Table 3), indicating that the 7-mer cocktail has a lower amplification bias than the MIG-seq cocktail.

**Table 3:** Percentage of called bases stratified by coverage depth.

Primer	Individual	Percentage of the medaka genome *1						
		Depth ≥ 1	≥ 3	≥ 10	≥ 30	≥ 100	≥ 500	≥ 1000
7-mer cocktail	Kun09	5.358	2.434	0.974	0.391	0.111	0.0146	0.00159
7-mer cocktail	Kun10	5.145	2.390	0.967	0.389	0.111	0.0144	0.00161
7-mer cocktail	Kun12	5.047	2.367	0.956	0.387	0.111	0.0147	0.00170
MIG-seq	Kun09	1.937	0.851	0.409	0.206	0.077	0.0192	0.00518
MIG-seq	Kun10	1.899	0.832	0.396	0.199	0.075	0.0186	0.00533
MIG-seq	Kun12	1.735	0.793	0.391	0.200	0.076	0.0192	0.00546

*1: Values shown are the called bases divided by the total base number, i.e., 734,040,372 bases, in the medaka genome. Each sample was extracted and adjusted to six million reads from the uniquely mapped reads in the BAM file.

Using the 7-mer primer cocktail (Table 1), 67 individuals from 15 wild populations and three artificial strains of medaka were subjected to MAAS analysis and 124,556 bases (genome coverage = 0.02%) were commonly sequenced with a depth ≥ 10 in all individuals. Figure 2d shows the reduction in the commonly sequenced bases along with the number of sampled individuals. The commonly sequenced bases reduced up to 10 individuals dramatically, after which the reduction was plateaued. Finally, we obtained 6479 SNVs with no missing individuals. After removing SNVs that were in linkage disequilibrium (r^2^ > 0.1) with another in each of the 50,000 base windows, 2987 SNVs were used for the population structure and admixture analyses. The obtained SNVs covered the entire genome (Fig. S1). Although the SNVs may not be uniformly distributed, there seems to be no strong bias.

To test the case when a reference genome is not available, we also conducted genotyping using de-novo assembled sequences using Stacks ver. 2.2^36^. De-novo assembled sequences were constructed using all the data for 67 individuals with the optimized mismatch threshold (ustacks –M 7). As results, 40,666 bases (genome coverage = 0.007%) were commonly sequenced and the number of SNVs was 1362 (Tables S3 and S4). Even with the reduced number of SNVs, the population structure based on the phylogeny could be reconstructed (Fig. S2).

### Population structure of wild populations and the origin of an artificial strain, Himedaka

**Figure 3:**
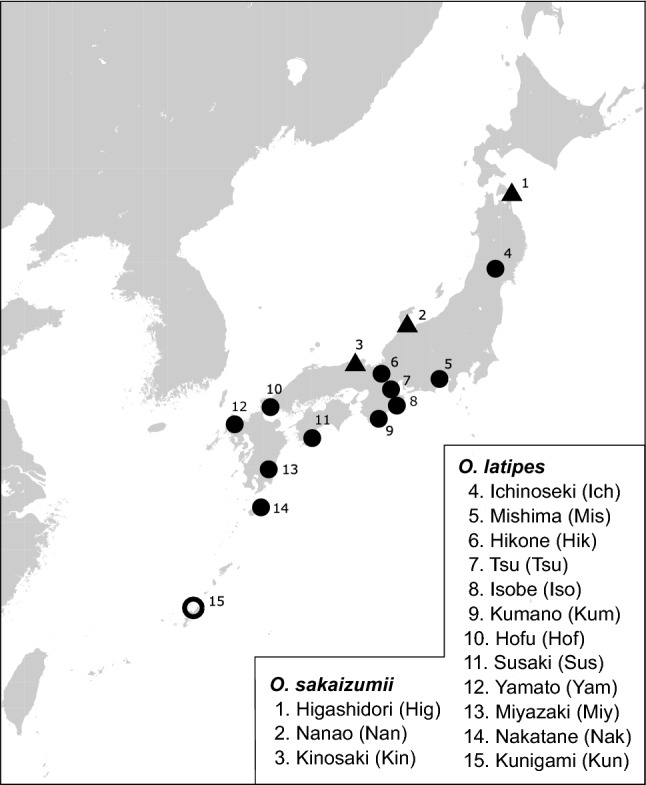
Geographic locations of collection sites. Filled circles and triangles on the map represent *O. sakaizumii* and *O. latipes*, respectively. The open circle represents a new collection site for wild medaka. The numbers and abbreviations correspond to the names in Table S11. The map was generated by GMT, Generic Mapping Tools ver.5.3.1.

**Figure 4:**
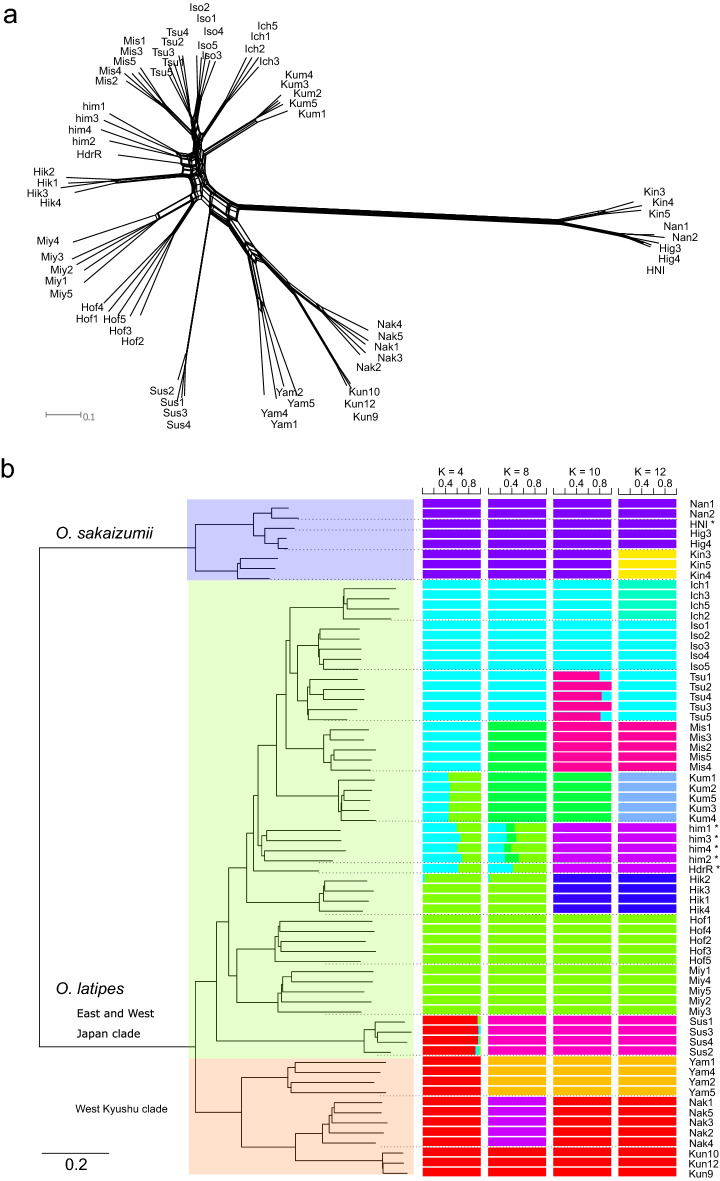
(**a**): Neighbor-net phylogenetic networks based on the 2987 SNPs; (**b**): Neighbor-joining tree and results of ADMIXTURE analysis for K = 4, 8, 10, 13. Asterisks indicate the artificial strains (Himedaka: him, HNI, HdrR). For the abbreviations of the wild populations, see Fig. 3 and Table S11.

The phylogenetic network and the neighbor-joining tree, as well as *D*_*XY*_ and *FST* values, suggested that *O. sakaizumii* and *O. latipes* are genetically well differentiated (Figs. 3, 4, Tables S5, S6). In *O. latipes*, a southwest to northeast genetic cline was roughly observed. The populations distributed in western Kyushu and the Nansei Islands, i.e., Kunigami, Nakatane and Yamato populations, formed a cluster, while the other populations formed another cluster (Fig. 4). The observed phylogeny was consistent with previous phylogeographic patterns observed in medaka using mitochondrial DNA sequences and nuclear SNVs^10,29^.

**Figure 5:**
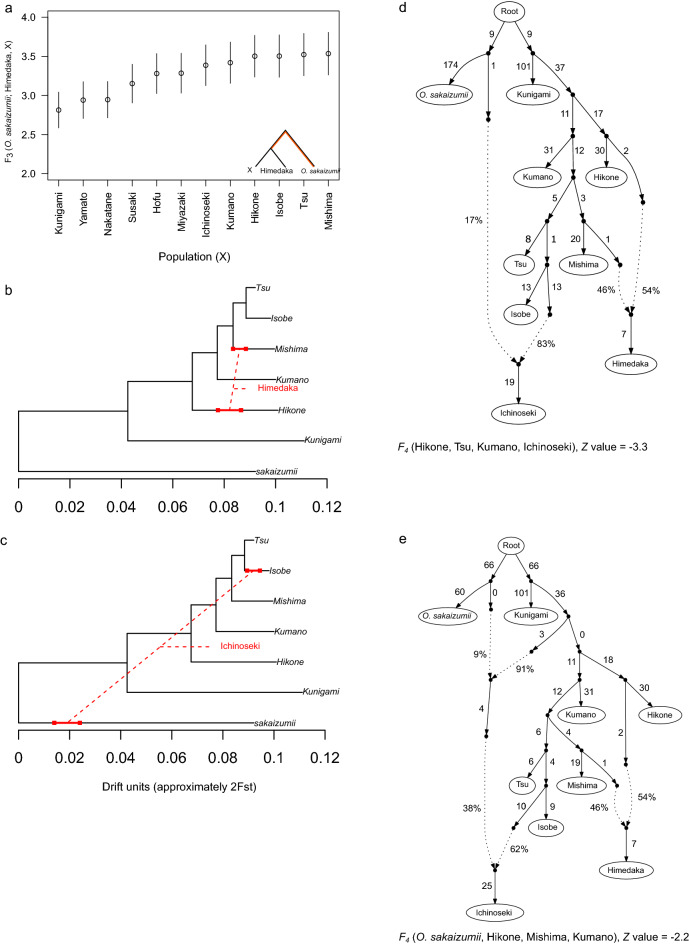
Admixture analyses focusing on the Himedaka and Ichinoseki populations. (**a**): Outgroup F_3_ statistics confirm which population is most closely related to Himedaka using *O. sakaizumii* as the outgroup. (**b**, **c**): Drift tree of unadmixed and admixed populations, where we assumed that the Himedaka strain (**b**) and Ichinoseki population (**c**) as hybrid populations. Tree branches, branches for drift following admixture, and range of the pre-split position were estimated by MixMapper (see Tables S6, S8). (**d**, **e**): Admixture graphs that fit the data. The branch length of the F_2_ drift distance is represented by the solid line and admixture events are represented by dotted lines with mixture proportions as shown.

Although the mitochondrial DNA haplotype of the Himedaka strain has been studied previously^29,33^, this study is the first to demonstrate the nuclear genomic characteristics of this strain. The neighbor-joining tree and the ancestral proportions estimated by the ADMIXTURE^37^ analysis indicated that the inbred strains Hd-rR and Himedaka, both of which have an orange phenotype, are closely related to each other (Fig. 4). The minimum value of the cross-validation errors in the ADMIXTURE analyses was observed at the number of the ancestral populations (*K*) = 12 (Fig. 4b, Table S7). The ADMIXTURE analysis also suggested that the Himedaka strain had been formed by the admixture of several wild populations (Fig. 4b, K = 4, 8). Outgroup *F*_*3*_ statistics showed that the Mishima population was most closely related to the Himedaka strain (Fig. 5a). To estimate which wild populations are likely to be the source of admixture in Himedaka, we performed MixMapper^38^ analysis. The results suggested that the Himedaka strain was formed by the admixture of the ancestors of the Mishima and Hikone populations (Fig. 5b); indeed, more than half of the bootstrap replicates in MixMapper supported this scenario (66/100, Table S8). The admixture graph based on a similar scenario also showed that the admixture proportions were 46% from a sister lineage of the Mishima population and 54% from a sister lineage of the Hikone population (Fig. 5d).

In this study, we sampled only several wild populations and, therefore, populations more closely related to the Himedaka strain may be found elsewhere. Even so, our study clearly demonstrated that the Himedaka strain was formed by crossing between different wild populations. Anthropogenic introductions of the commercially available Himedaka strain could potentially disrupt the genetic structure of local populations^29,34^. Therefore, our results will be useful in quantitative assessments of genetic disturbance of wild populations by this commercially available strain.

We also examined the possibility of genetic admixture between *O. latipes* and *O. sakaizumii* in wild populations. To date, the natural hybrids between *O. latipes* and *O. sakaizumii* have been reported in the Tango-Tajima area, which is a coastal area on the Sea of Japan in Kyoto and Hyogo prefectures^10,14^. Similarly, there are several known sites in eastern Honshu where *O. sakaizumii* and *O. latipes* mitochondrial haplotypes are co-localized^29,39^. Although previous studies have presumed that the extant distributions are the result of recent anthropogenic introductions^29,39^, no attempt has been made to conduct admixture analyses of populations in eastern Honshu.

We applied the *ABBA-BABA* test to detect admixture among wild populations. We examined *D*(X_1_, X_2_; Kunigami, *O. sakaizumii*), where X_1_ and X_2_ are Ichinoseki, Isobe, Tsu, or Mishima populations, and found that the absolute Z values increased (|Z|= 1.81–1.97) when the Ichinoseki population was included (Table S9). Therefore, we assumed that the Ichinoseki population is the result of hybridization between populations of *O. latipes* and *O. sakaizumii*. MixMapper results also supported these findings (Table S10, Figs. 5c,d, S3). Although we found evidence of gene flow from *O. sakaizumii* to the Ichinoseki population, this gene flow may have occurred indirectly thorough another population (Figs. 5e, S3). Such a scenario may account for the observation of a smaller maximum |Z| value (Fig. 5e, |Z|= 2.2) compared to those observed in populations with direct gene flow from *O. sakaizumii* (Fig. 5d; |Z|= 3.3). In addition, Fig. 5d shows inconsistent drift parameters between *O. sakaizumii* and *O. latipes* lineages before the admixture occurs, suggesting that a scenario in which there is indirect gene flow (Fig. 5e).

Population genetics based on nuclear SNV data are indispensable for identifying admixture events, including natural hybridization and anthropogenic introduction. To more fully understand the natural history of wild medaka populations and the impact of anthropogenic introductions, further comprehensive sampling is needed. The method developed in this study will be useful for future population genomics studies on medaka and on other organisms.

### Concluding remarks

We successfully sequenced an average of one million bases with depth ≥ 10 per million reads using our developed MAAS method. The sequencing efficiency per read was improved by two- to three-fold compared to previous methods. We obtained 2987 informative SNVs with no missing genotype calls for 67 individuals from wild populations and artificial strains, which was sufficient for estimating population genetic structure. Using the obtained SNVs, we performed admixture analyses and identified possible source populations for the artificial Himedaka strain. In addition, we also detected the signature of admixture between *O. sakaizumii* and *O. latipes* in a population from eastern Honshu. We consider that MAAS will be a useful tool for examining the population structure of taxa and for reconstructing the admixture history of populations.

## Materials and methods

### DNA sample collection

To analyze the population structure of wild medaka populations, we selected samples from the DNA collection of Takehana et al.^29^, deposited in University of Shizuoka. The original DNA collection had been made throughout 1980s and 2000s. The selected samples covered the major mitotypes and contained more than three individuals of each population (Table S11, Fig. 3), which were collected from three collection sites for *O. sakaizumii* and 12 collection sites for *O. latipes*. We also examined several artificial strains: HNI and Hd-rR, which are inbred strains derived from *O. sakaizumii* and *O. latipes*, respectively, and four Himedaka individuals from commercial stock (Uruma city, Okinawa Prefecture, Japan).

In addition, samples were newly collected at Kunigami Village, Okinawa Prefecture. Live fish were anesthetized with MS-222 (aminobenzene methanesulfonate, FUJIFILM Wako Pure Chemical Corporation, Osaka, Japan) and then fixed in 99% ethanol. Genomic DNA was extracted using a DNeasy kit (Qiagen Inc., Hilden, Germany) from ethanol-fixed pectoral fin samples according to the manufacturer’s protocol. The DNA concentration was measured using a spectrophotometer (Nanodrop 1000, Thermo Fisher Scientific, Waltham, Massachusetts, USA), and the DNA was diluted with PCR-grade water to a concentration of c.a. 10 ng/µl (UltraPure™ DNase/RNase-Free Distilled Water, Thermo Fisher Scientific).

### Ethic statement

All methods were carried out in accordance with the Regulation for Animal Experiments at University of the Ryukyus for handling live fish. All experiments were approved by the Animal Care Ethics Committee of University of the Ryukyus (R2019035). All experimental methods are reported in accordance with ARRIVE guidelines.

### PCR primer design

The following steps were used to select primers for MAAS (Fig. 1). (1) All possible 10-mer sequence combinations (i.e., 4^10^ = 1,048,576 sequences) were generated in silico. (2) The sequences containing simple sequence repeats, some of which had been used in the MIG-seq method^17^, were excluded. (3) Sequences containing a functional motif, such as a transcription factor-binding site, were also excluded because they may not be suitable for examining neutral genetic markers. We obtained a catalog of motifs from the JASPAR CORE^40^ (http://jaspar.genereg.net). (4) To avoid taxon-dependency in primer performance, we used information about the *k*-mer (k = 10) frequency of reference genomes from multiple phyla. Sequences that showed marked differences in frequency among taxa were excluded. The frequencies of each 10-mer sequence in the reference genomes of 17 species belonging to 12 phyla of metazoa were counted (Table S12) using the “oligonucleotideFrequency” function in the “Biostrings” package ver. 2.4^41^. In each of these taxa, the frequencies of sequences were stratified into three grades (< 10^2^, 10^2^–10^3^, and > 10^3^). We then selected the sequences that showed the same grade in more than 80% (14/17) of the species. (5) To avoid synthesizing primer dimers, self-complementary sequences were excluded, taking Illumina adapter sequences (5′-CGCTCTTCCGATCT-3′ and 5′-TGCTCTTCCGATCT-3′) into account. Self-complementation of two bases at the 3′-end or every three continuous bases in primer sequences was then evaluated using a custom script in R ver. 3.5.0 (R Development Core Team, http://cran.r-project.org). Based on the selected 10-mer sequences (i.e., 129 sequences, Fig. 1), 7-mer primer sequences were designed by removing the 3 bases at the 3' end. Finally, we selected 24 candidate sequences for both 10-mer and 7-mer primers for the subsequent step (Table S1).

The primer sequence consisted of three parts^17^: partial sequence of the Illimina adapter, 7 N bases, and a short priming sequence, e.g., 5′-CGCTCTTCCGATCTNNNNNNNGTCGCCC-3′. PCR amplification was performed using the candidate primers using the first PCR protocol described below (Table S1). Banding patterns were observed by electrophoresis on 1% agarose gels (agarose S; TaKaRa, Japan). Of the candidate primers, we selected four 7-mer primers and four 10-mer primers that each gave a smeared banding pattern with amplification products ranging from 500 to 2000 bp, indicating uniform amplification of multiple target sequences (Table S1).

### Library construction and sequencing

The library was constructed by a two-step PCR approach using a modification of a MIG-seq protocol^14^. In the first PCR step, multiple regions of genomic DNA were amplified using a cocktail of primers with a Multiplex PCR Assay Kit Ver.2 (TaKaRa) (Table 1). The volume of the PCR reaction mixture was 7 μl, containing 1 μl of template DNA, 2 μM of each PCR primer, 3.5 μl of 2 × Multiplex PCR Buffer, and 0.035 μl of Multiplex PCR Enzyme Mix. PCR was performed under the following conditions: denaturation at 94 °C for 1 min; 25 cycles of 94 °C for 30 s, 38 °C for 1 min, and 72 °C for 1 min, followed by a final extension step at 72 °C for 10 min.

The primers in the second PCR step contained the Illumina sequencing adapter and an index sequence to identify each sample. Following the Truseq indexes, we used the combinations of eight forward indexes (i5) and 12 reverse indexes (i7), which resulted in a total of 96 combinations. To be used as a template for the second PCR, the first PCR product from each sample was diluted 50 times with PCR-grade water. The second PCR was performed in a 15-μl reaction mixture containing, 3 μl of diluted first PCR product, 3 μl of 5 × PrimeSTAR GXL Buffer, 200 μM of each dNTP, 0.2 μM of forward index primer and reverse index primer, 0.375 U of PrimeSTAR GXL DNA Polymerase (TaKaRa). The PCR conditions were as follows: 12 cycles at 98 °C for 10 s, 54 °C for 15 s, and 68 °C for 30 s.

The second PCR product of each sample was pooled by equal volume and size-selected from 600 to 1000 bp using solid phase reversible immobilization (SPRI) select beads (Beckman Coulter Inc, Brea, California, USA) according to the manufacturer’s protocol. The DNA concentration of the pooled library was measured using a Qubit fluorometer (Thermo Fisher Scientific). We sequenced the libraries using two NGS platforms, MiSeq (Illumina, MiSeq Reagent Kit v2 Micro, Paired-End (PE), 150 bp) and HiSeq X (Illumina, PE, 150 bp). Sequencing using the HiSeq X platform was performed by Macrogen Japan (Tokyo, Japan).

To compare primer performance, the DNA libraries constructed using the 7-mer and 10-mer primers for one individual were sequenced using MiSeq. Then, a 7-mer primer cocktail containing four sets of mixed primers was used for the subsequent analyses (Table 1). We also constructed DNA libraries using 7-mer and MIG-seq primer cocktails for three individuals and sequenced them using the HiSeq X platform. Finally, we constructed DNA libraries using 7-mer primer cocktails for 67 wild individuals and six artificial strain individuals for population genetics analyses (Table S11, Fig. 3).

### Mapping and SNV calling

Genotyping was conducted using the following BWA-GATK best-practices pipeline for each sample^42^. Primer sequences were removed using cutadapt with the –b option selected^43^. The Illumina adapter sequences were also removed and quality filtering was performed using fastp ver. 0.20.0 with the “–detect_adapter_for_pe, –cut_front” option selected^44^. The remaining reads were mapped on the reference genome of medaka, Hd-rR strain, GCA_002234675.1; ASM223467v1^27^ using Burrows-Wheeler Alignment tool, BWA mem ver. 0.7.17^45^. After mapping, output files were converted to Binary Alignment/Map (BAM) format using SAMtools ver. 1.7^46^. SNVs and InDels in the sample were determined following the best practice guidelines set out in the Genome Analysis Tool Kit (GATK ver. 3.8.0)^42^. We then filtered out SNVs and InDels based on the following criteria: “QD < 2.0 || FS > 60.0 || MQ < 40.0 || MQRankSum < −12.5 || ReadPosRankSum < −8.0” for SNVs and “QD < 2.0 || FS > 200.0 || ReadPosRankSum < −20.0” for InDels. Based on the filtered SNVs and InDels, we recalibrated the mapping quality of each read and extracted sequences including variant and non-variant sites in vcf format using the “GenotypeGVCFs” command in GATK with the “–includeNonVariantSites” option selected.

### Sequencing performance evaluation

To compare genotyping performance between primer cocktails (7-mer and MIG-seq), we evaluated the performance based on four indicators. (1) The proportion of properly sequenced reads were evaluated, which was taken as the read number in the raw fastq data that passed quality filtering with ‘fastp’; (2) The proportion of reads uniquely mapped on the reference genome was calculated. To extract uniquely mapped reads from the BAM file, we used the “samtools view” command with the “-q 1-F 4-F 256” option selected and grep with “-v -e XA:Z-v-e SA:Z”. (3). The sequencing output in genotyping should be evaluated after standardizing by read number. We extracted and analyzed 6 million reads from the uniquely mapped reads in each sample. The distribution of the coverage depth in the sequenced positions was then examined to evaluate the amplification bias. We stratified the depth thresholds (≥ 1, ≥ 3, ≥ 10, ≥ 30, ≥ 100, ≥ 500 and ≥ 1000) and calculated the proportion of each group for all of the bases in the reference genome (734,040,372 bp); (4) The saturation curve of the number of reads and the sufficiently sequenced bases was then calculated. To do this, we extracted subsets of the reads (0.5, 1, 2, and 6 million reads) from the original BAM file and considered only those sequences with depths of more than 10 using bcftools ver.1.7 (http://samtools.github.io/bcftools/); (5) The reduction curve of the commonly sequenced bases among randomly chosen individuals of *O. latipes* was then estimated using the following equation with the “nls” function in R: (C_1_ = 2,051,200, C_2_ = 325,000, α = 0.71):

$$Y = C_{1} \times a^{x} + C_{2}$$

where Y is the total number of bases with no missing genotype calls, x is the number of individuals, α, C_1_ and C_2_ is the constants. Since a reference genome is often not available for population genetic analyses involving non-model organisms, we also performed de novo assembly and genotyping without the reference genome to simulate such a situation. De novo assembly and genotyping were conducted using “denovo_map.pl” in Stacks ver. 2.2^36,47^ using the fastq of the 67 individuals, which were consistent with the population structure analysis described below in the next section. The parameter in the de-novo assembly was optimized following the manual^47^. We ran several iterations with different parameter (“ustacks –M”, 1–9). The optimized parameter was chosen as M = 7, which maximized the number of polymorphic loci where more than 80% of the samples were genotyped.

### Analysis of population structure and admixture

The SNV data were prepared using bcftools and VCFtools ver.0.1.15 (http://vcftools.sourceforge.net/). For each individual, the sequenced positions with depths ≥ 10 were included in the analysis. Due to low read numbers and genotyped bases, eight individuals were excluded from the analysis (Table. S11). The individual vcf files were merged into a multi-sample vcf file and the sequence positions that were genotyped in all individuals were extracted using the “bcftools view” command with the option “NO_MISSING = 0”. As a result, 126,765 bases were sequenced including non-variant and variant positions from 67 individuals (8 *O. sakaizumii* and 59 *O. latipes*), and 6479 SNV and InDel sites were identified. Based on the total sequenced bases, we calculated nucleotide diversity *π* and pairwise genetic distances *D*_*XY*_. Weighted *FST* values of wild populations were also calculated using pixy ver. 1.0.0^48^. Then, we excluded sites with three or more alleles and InDels. We also removed SNVs that showed strong linkage disequilibrium (r^2^ > 0.1) in each of the 50,000 base windows using plink ver. 1.90^49^. Sequences were deposited in the DDBJ Sequence Read Archive (DRA) database under the accession number DRA011342.

After filtering, 2987 SNVs remained. Phylogenetic relationships were then estimated by the neighbor-joining method using a genetic distance matrix by the “snpgdsDiss” function implemented in the “SNPRelate” package ver. 1.22.0^50,51^. The distance matrix was converted to the nexus format using the “writeDist” function of the “phangorn” package ver. 5.0^52,53^. The phylogenetic tree and network were drawn using SplitsTree ver. 4.15.1^54^ and Figtree ver. 1.4.3 (https://github.com/rambaut/figtree/). The branch between *O. latipes* and *O. sakaizumii* was considered as the root. To estimate the population structure, we used ADMIXTURE ver. 1.3.0^37^. ADMIXTURE was run for the number of clusters (*K*) from 1 to 15. Statistical support for the different number of clusters was evaluated based on the lowest cross-validation errors. We ran 50 replicates with random seeds for each *K* and calculated the mean of the cross-validation errors^10^ (Table. S7).

### Reconstruction of admixture among wild populations and artificial strains

Himedaka is considered to be a cross-breeding strain derived from several wild populations^55^. To elucidate the genetic background of strains and populations, we used the *F* statistics framework^24,56^. To identify populations that are closely related to Himedaka, we used outgroup *F*_*3*_ statistics with the “qp3Pop” command in Admixtools ver. 7.0^56^. We also performed an *ABBA-BABA* test and calculated Patterson’s D statistics using “qpDstat”^24,56^, which estimates admixture among four populations. For this analysis, SNVs that were polymorphic in the four populations were extracted using the “admixr” package^57^.

We reconstructed the admixture graph under an assumed demographic scenario using “qpGraph” command in the Admixtools^24,56^. We selected the model in which the *F*_*4*_ statistics for all the population combinations were less than three. To estimate the source populations of admixture, we performed analyses using MixMapper ver. 2.0^38^. MixMapper semi-automatically detects source populations of admixture when we specify the number of admixture events and the target of the admixed population.

## Supplementary Information

Supplementary Information.

## Data Availability

Genetic data: Raw sequence reads are deposited in the DDBJ Sequence Read Archive (DRA) database (DRA011342). Individual genotype data are available on Dryad (Reviewer URL: https://datadryad.org/stash/share/t5mLTLw0nfGzHlMpqUYsC8MdHoz2aL67ane5I0bWpIA).

## References

[1] Andrews KR, Good JM, Miller MR, Luikart G, Hohenlohe PA (2016). Harnessing the power of RADseq for ecological and evolutionary genomics. Nat. Rev. Genet..

[2] Rohland N, Reich D (2012). Cost-effective, high-throughput DNA sequencing libraries for multiplexed target capture. Genome Res..

[3] Craig DW (2008). Identification of genetic variants using bar-coded multiplexed sequencing. Nat. Methods.

[4] Peterson, B. K., Weber, J. N., Kay, E. H., Fisher, H. S. & Hoekstra, H. E. Double digest RADseq: An inexpensive method for de novo SNP discovery and genotyping in model and non-model species. *PLoS ONE* (2012). 10.1371/journal.pone.0037135 PMC3365034 22675423

[5] Shah N (2020). Extreme genetic signatures of local adaptation during Lotus japonicus colonization of Japan. Nat. Commun..

[6] Machado HE (2016). Comparative population genomics of latitudinal variation in *Drosophila simulans* and *Drosophila melanogaster*. Mol. Ecol..

[7] Malinsky M (2018). Whole-genome sequences of Malawi cichlids reveal multiple radiations interconnected by gene flow. Nat. Ecol. Evol..

[8] Liu S, Hansen MM, Jacobsen MW (2016). Region-wide and ecotype-specific differences in demographic histories of threespine stickleback populations, estimated from whole genome sequences. Mol. Ecol..

[9] Baris TZ (2017). Evolved genetic and phenotypic differences due to mitochondrial-nuclear interactions. PLOS Genet..

[10] Katsumura T, Oda S, Mitani H, Oota H (2019). Medaka population genome structure and demographic history described via genotyping-by-sequencing. G3: Genes Genomes Genet..

[11] Elshire RJ (2011). A robust, simple genotyping-by-sequencing (GBS) approach for high diversity species. PLoS ONE.

[12] Graham CF (2015). Impacts of degraded DNA on restriction enzyme associated DNA sequencing (RADSeq). Mol. Ecol. Resour..

[13] Campbell NR, Harmon SA, Narum SR (2015). Genotyping-in-Thousands by sequencing (GT-seq): A cost effective SNP genotyping method based on custom amplicon sequencing. Mol. Ecol. Resour..

[14] Takehana Y (2016). Origin of boundary populations in Medaka (*Oryzias latipes* Species Complex). Zool. Sci..

[15] Bayerl H (2018). Fast and cost-effective single nucleotide polymorphism (SNP) detection in the absence of a reference genome using semideep next-generation Random Amplicon Sequencing (RAMseq). Mol. Ecol. Resour..

[16] Hosoya S (2019). Random PCR-based genotyping by sequencing technology GRAS-Di (genotyping by random amplicon sequencing, direct) reveals genetic structure of mangrove fishes. Mol. Ecol. Resour..

[17] Suyama, Y. & Matsuki, Y. MIG-seq: An effective PCR- based method for genome-wide single-nucleotide polymorphism genotyping using the next- generation sequencing platform. *Sci. Rep.* (2015). 10.1038/srep16963 PMC4655332 26593239

[18] Caetano-Anollés G (1994). MAAP: A versatile and universal tool for genome analysis. Plant Mol. Biol..

[19] Takahashi Y (2016). Lack of genetic variation prevents adaptation at the geographic range margin in a damselfly. Mol. Ecol..

[20] Eriksson CE, Ruprecht J, Levi T (2020). More affordable and effective noninvasive single nucleotide polymorphism genotyping using high-throughput amplicon sequencing. Mol. Ecol. Resour..

[21] Städele V, Linda V (2016). Strategies for determining kinship in wild populations using genetic data why determine kinship in Wild. Ecol. Evol..

[22] Watanabe K (2020). Large-scale hybridization of Japanese populations of Hinamoroko, Aphyocypris chinensis, with A. kikuchii introduced from Taiwan. Ichthyol. Res..

[23] Green RE (2010). A draft sequence of the neandertal genome. Science.

[24] Peter BM (2016). Admixture, population structure, and f-statistics. Genetics.

[25] Martin SH, Davey JW, Jiggins CD (2015). Evaluating the use of ABBA-BABA statistics to locate introgressed loci. Mol. Biol. Evol..

[26] Durand EY, Patterson N, Reich D, Slatkin M (2011). Testing for ancient admixture between closely related populations. Mol. Biol. Evol..

[27] Ichikawa, K. *et al.* Centromere evolution and CpG methylation during vertebrate speciation. *Nat. Commun.* (2017). 10.1038/s41467-017-01982-7 PMC5705604 29184138

[28] Iwamatsu T (2006). The Integrated Book For the Biology of the Medaka.

[29] Takehana Y, Nagai N, Matsuda M, Tsuchiya K, Sakaizumi M (2003). Geographic variation and diversity of the cytochrome b gene in Japanese wild populations of medaka, *Oryzias latipes*. Zool. Sci..

[30] Katsumura T (2009). Genetic differentiation among local populations of medaka fish (*Oryzias latipes*) evaluated through grid- and deme-based sampling. Gene.

[31] Asai T, Senou H, Hosoya K (2011). A new ricefish from northern Japan (Teleostei: Adrianichthyidae). Ichthyol. Explor. Freshw..

[32] Takehana Y, Uchiyama S, Matsuda M, Jeon S, Sakaizumi M (2004). Geographic variation and diversity of the cytochrome b gene in wild populations of Medaka (*Oryzias latipes*) from Korea and China. Zool. Sci..

[33] Koyama N, Mori T, Nakai K, Kitagawa T (2011). Genetic composition of commercial strains of *Oryzias latipes* revealed by mtDNA analyses, Japan. Jpn. J. Ichthyol..

[34] Nakao R, Iguchi Y, Koyama N, Nakai K, Kitagawa T (2017). Current status of genetic disturbances in wild medaka populations (*Oryzias latipes* species complex) in Japan. Ichthyol. Res..

[35] Horoiwa M (2022). Integrated population genomic analysis and numerical simulation to estimate larval dispersal of Acanthaster cf. solaris between Ogasawara and other Japanese Regions. Front. Mar. Sci..

[36] Rochette NC, Rivera-Colón AG, Catchen JM (2019). Stacks 2: Analytical methods for paired-end sequencing improve RADseq-based population genomics. Mol. Ecol..

[37] Alexander DH, Lange K (2011). Enhancements to the ADMIXTURE algorithm for individual ancestry estimation. BMC Bioinform..

[38] Lipson M (2013). Efficient moment-based inference of admixture parameters and sources of gene flow. Mol. Biol. Evol..

[39] Kitano S (2014). Non-native mitochondrial haplotype in wild Medaka population from Nagano prefecture. Rep. Nagano Prefect Inst. Environ. Conserv..

[40] Khan A (2018). JASPAR 2018: Update of the open-access database of transcription factor binding profiles and its web framework. Nucl. Acids Res..

[41] Pages H, Aboyoun P, Gentleman R, DebRoy S (2016). Biostrings: String objects representing biological sequences, and matching algorithms. R Packag. Vers..

[42] Van der Auwera GA (2013). From FastQ data to high confidence variant calls: The genome analysis toolkit best practices pipeline. Curr. Protoc. Bioinfom..

[43] Martin M (2011). Cutadapt removes adapter sequences from high-throughput sequencing reads. EMBnet. J..

[44] Chen S, Zhou Y, Chen Y, Gu J (2018). Fastp: An ultra-fast all-in-one FASTQ preprocessor. Bioinformatics.

[45] Li, H. Aligning sequence reads, clone sequences and assembly contigs with BWA-MEM. *arXiv* **1303**, (2013).

[46] Li H (2009). The sequence alignment/map format and SAMtools. Bioinformatics.

[47] Paris JR, Stevens JR, Catchen JM (2017). Lost in parameter space: A road map for stacks. Methods Ecol. Evol..

[48] Korunes KL, Samuk K (2021). pixy: Unbiased estimation of nucleotide diversity and divergence in the presence of missing data. Mol. Ecol. Resour..

[49] Purcell S (2007). PLINK: A tool set for whole-genome association and population-based linkage analyses. Am. J. Hum. Genet..

[50] Zheng X (2012). A high-performance computing toolset for relatedness and principal component analysis of SNP data. Bioinformatics.

[51] Weir BS, Goudet J (2017). A unified characterization of population structure. Genetics.

[52] Paradis E, Schliep K (2019). Ape 5.0: An environment for modern phylogenetics and evolutionary analyses in R. Bioinformatics.

[53] Schliep KP (2011). phangorn: Phylogenetic analysis in R. Bioinformatics.

[54] Huson DH, Bryant D (2006). Application of phylogenetic networks in evolutionary studies. Mol. Biol. Evol..

[55] Nakai K, Nakao R, Fukamachi S, Koyama N, Kitagawa T (2011). Genetic analysis of wild Medaka (*Oryzias latipes*) populations in the Yamato River, Nara Prefecture, Japan: Detection of the b allele responsible for the “himedaka” phenotype. Jpn. J. Ichthyol..

[56] Patterson N (2012). Ancient admixture in human history. Genetics.

[57] Petr M, Vernot B, Kelso J (2019). Admixr-R package for reproducible analyses using ADMIXTOOLS. Bioinformatics.

