# Supplementary material for: Population admixtures in medaka inferred by multiple arbitrary amplicon sequencing
Source: Sci Rep. 2022 Nov 21;12:19989. doi: 10.1038/s41598-022-24498-7 (PMC9678866; doi:10.1038/s41598-022-24498-7)
Supplement: Supplementary file 1 — Supplementary Information. [file 41598_2022_24498_MOESM1_ESM.pdf]

Supporting information

Population admixtures in medaka inferred by multiple arbitrary amplicon sequencing

Shingo Fujimoto*[1, 2, 7], Hajime Yaguchi[2, 3], Taijun Myosho[4], Hiroaki Aoyama[5, 6], Yukuto Sato[5, 7], Ryosuke Kimura*[1]

1 Graduate School of Medicine, University of the Ryukyus, Okinawa 903-0125, Japan

2 Tropical Biosphere Research Center, University of the Ryukyus, Okinawa 903-0213, Japan

3. Department of Bioscience, School of Science and Technology, Kwansei Gakuin University, Sanda, Hyogo, 669-1330, Japan (Present address)

4 Laboratory of Molecular Reproductive Biology, Institute for Environmental Sciences, University of Shizuoka, Shizuoka, 422-8526, Japan

5 Center for Strategic and Research Center, University of the Ryukyus, Okinawa 903-0213, Japan

6 Research Planning Office, University of the Ryukyus, Okinawa 903-0213, Japan

7 Research Laboratory Center, Faculty of Medicine, University of the Ryukyus, Okinawa 903-0213, Japan (Present address)

* Corresponding authors

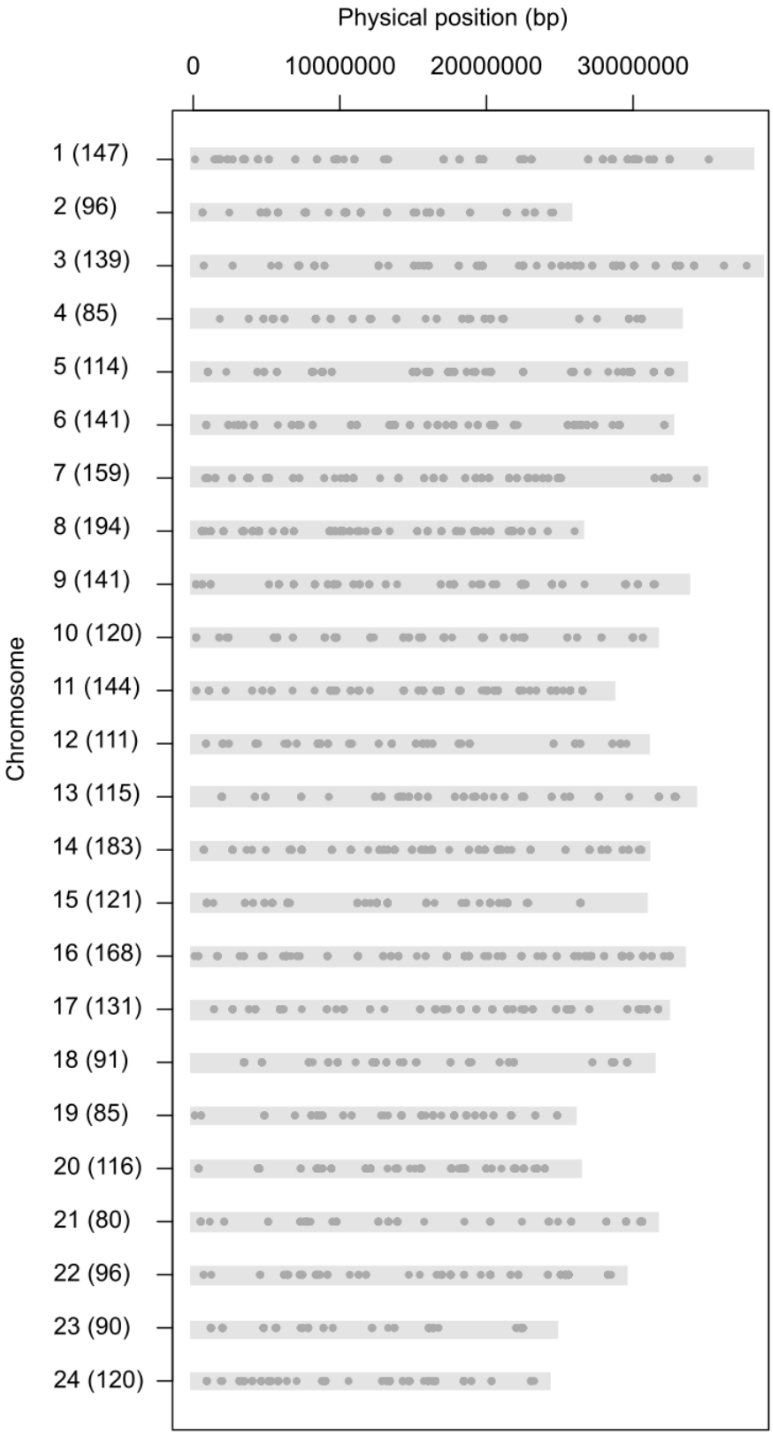

Figure. S1. The distribution of 2987 SNVs on chromosome used for population structure analysis. Numbers in parentheses indicate SNVs on the chromosome. The gray area represented each chromosome's length

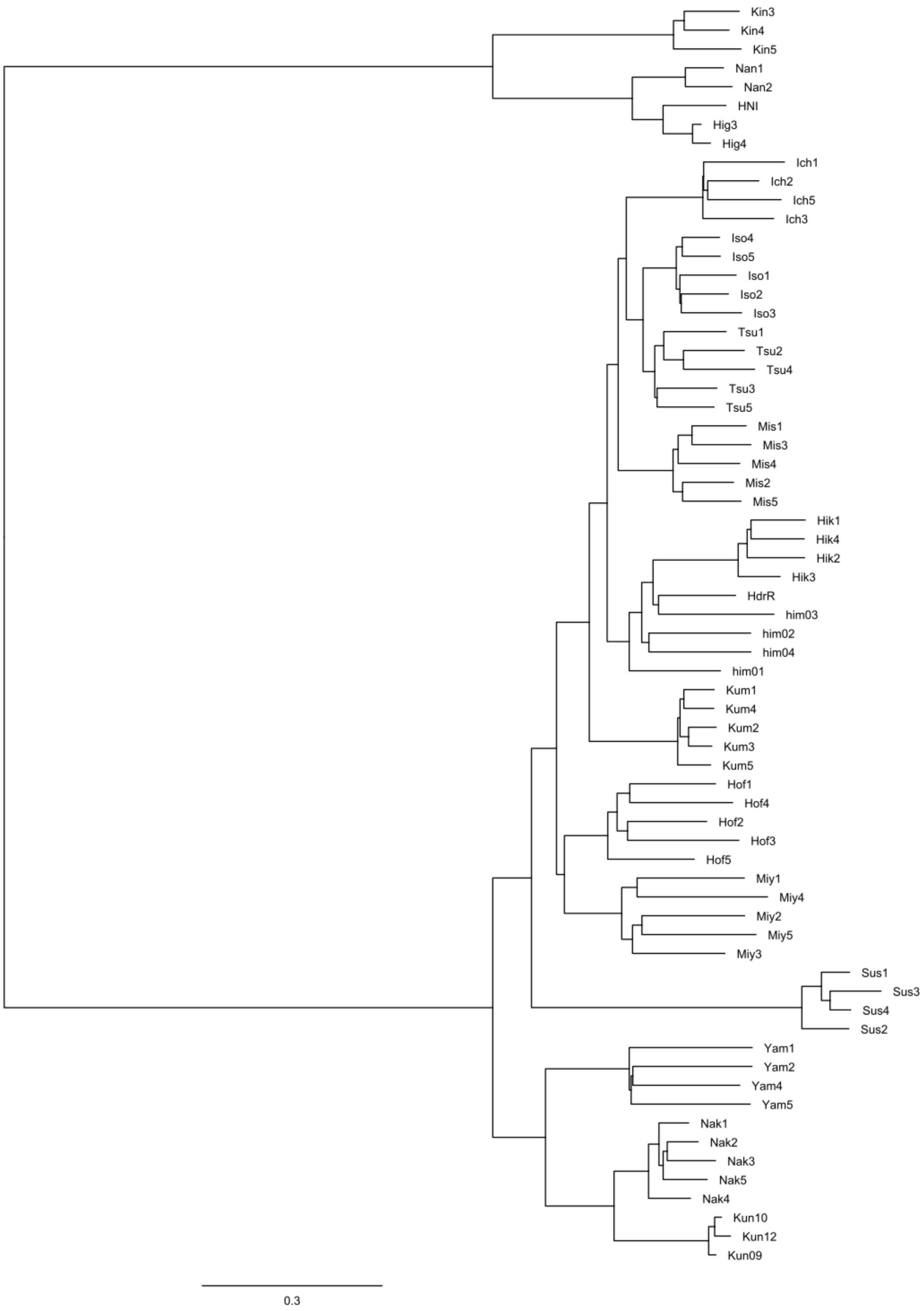

Figure. S2. Neighbor-joining tree estimated for the 1362 SNVs obtained using stacks without the reference genome. *Oryzias sakaizumii* was used as an outgroup.

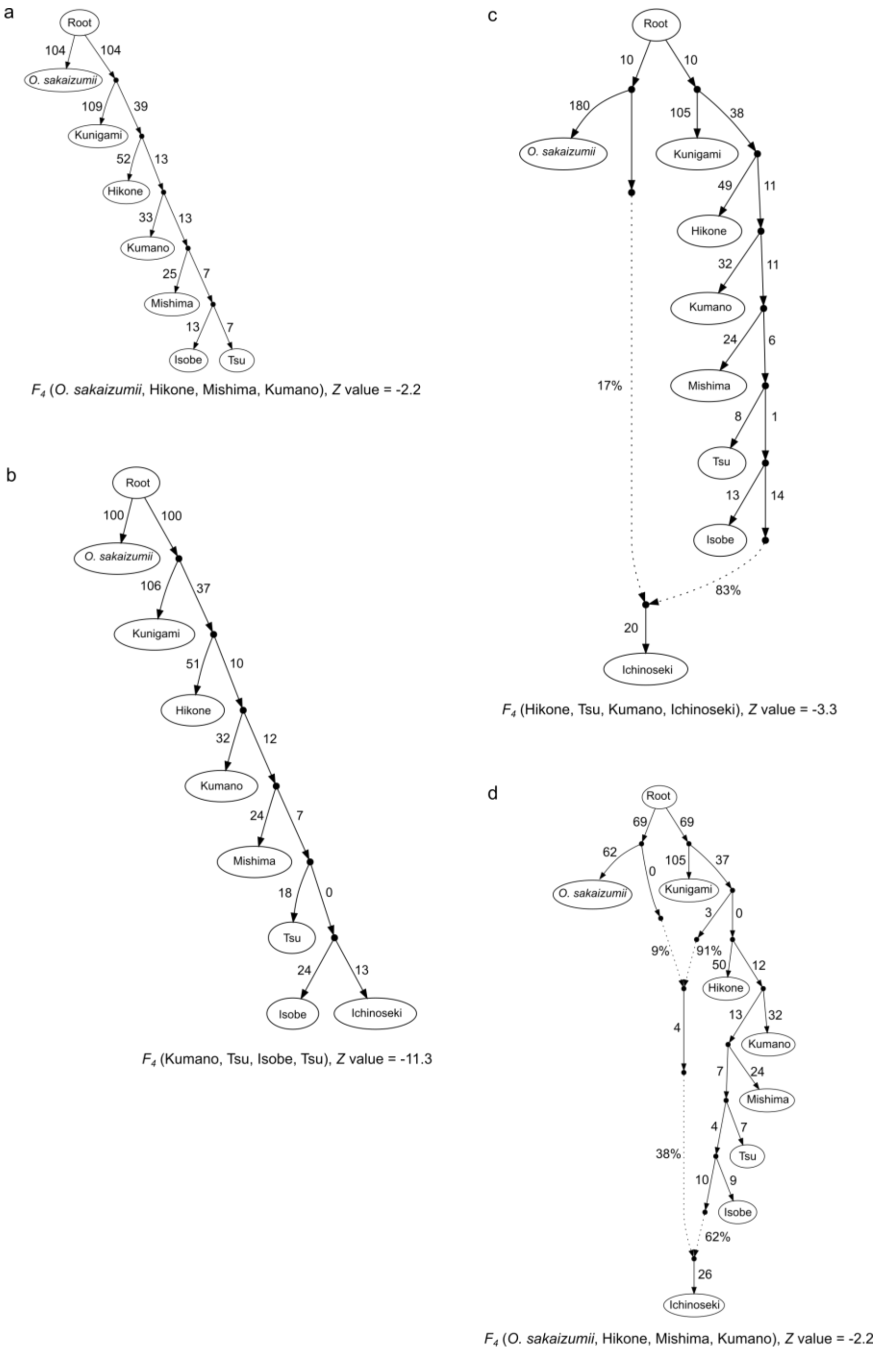

Figure S3. Admixture graphs focusing on the admixture events involving the Ichinoseki population. (a-b) Drift tree without (a) and with (b) the Ichinoseki population. (c) Admixture event between *O. sakaizumii* and the Ichinoseki population. (d) Admixture event between *O. latipes* populations. The branch length of the F_2 drift distance is represented by the solid line and admixture events are represented by dotted lines with mixture proportions as shown.

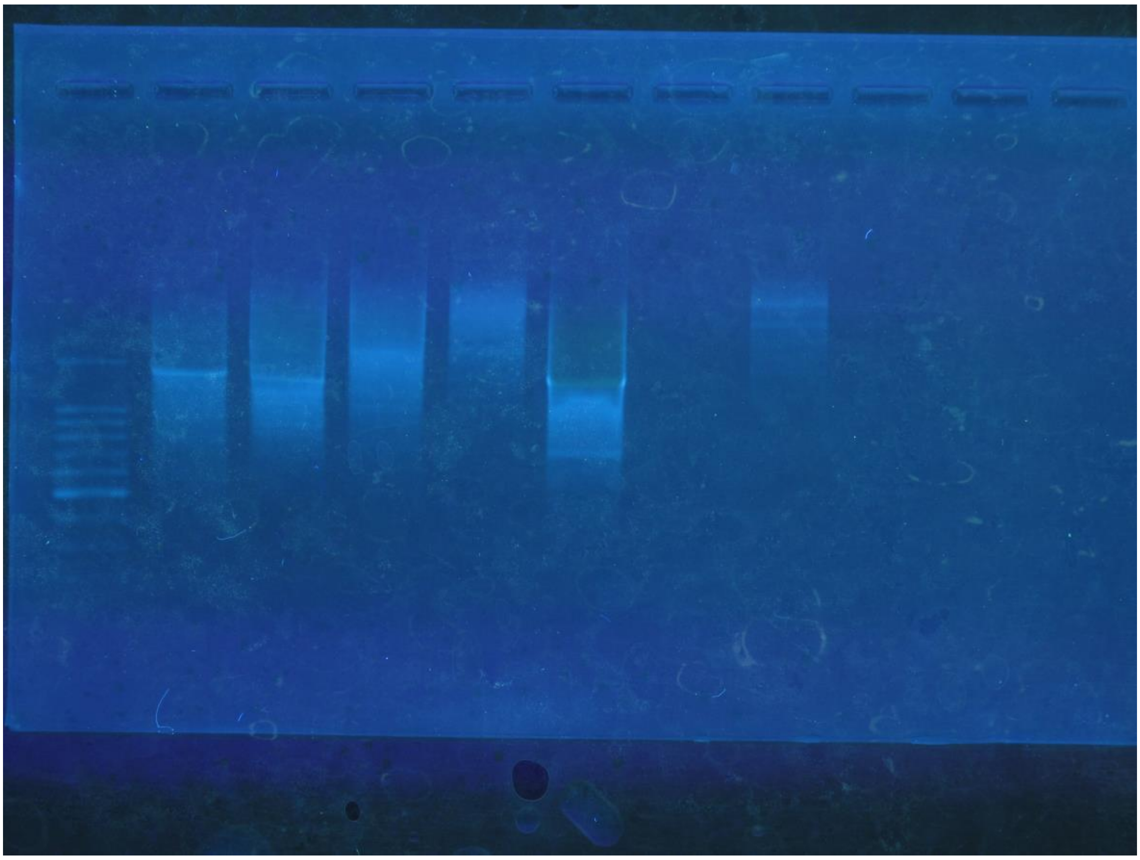

Figure. S4. Original picture of gel electrophoresis image in Fig. 2A

Table S1. Collection site, mitochondrial DNA genotype and number of samples

Species	Collection site / Strain	Abbreviation	mtDNA subclade *1	Mitotype *2	Number genotyped	Number analyzed *3	Source of sample
O. latipes	Kunigami	Kunigami	B-XI	B24*(2)	3	3	Wild collection (This study)
O. latipes	Yamato	Yam	B-XI	B24*(5)	5	4	Wild collection (Takehana et al. 2003; Table 1, site 258)
O. latipes	Nakatane	Nak	B-VIII	B8*(5)	5	5	Wild collection (Takehana et al. 2003; Table 1, site 293)
O. latipes	Miyazaki	Miy	B-VIII	B4*(5)	5	5	Wild collection (Takehana et al. 2003; Table 1, site 279)
O. latipes	Hofu	Hof	B	B13*(5)	5	5	Wild collection (Takehana et al. 2003; Table 1, site 227)
O. latipes	Susaki	Sus	B-V	B1d*(5)	4	4	Wild collection (Takehana et al. 2003; Table 1, site 245)
O. latipes	Hikone	Hik	B-VII	B22*(5)	4	4	Wild collection (Takehana et al. 2003; Table 1, site 158)
O. latipes	Kumano	Kum	B-VI	B33*(5)	5	5	Wild collection (Takehana et al. 2003; Table 1, site 155)
O. latipes	Tsu	Tsu	B-II	B1c(5)	5	5	Wild collection (Takehana et al. 2003; Table 1, site 147)
O. latipes	Isobe	Iso	B	B1c(1), B5(3), B11(1)	5	5	Wild collection (Takehana et al. 2003; Table 1, site 152)
O. latipes	Mishima	Mis	B-IV	B18*(5)	5	5	Wild collection (Takehana et al. 2003; Table 1, site 136)
O. latipes	Ichinoseki	Ich	B	B11(5)	5	4	Wild collection (Takehana et al. 2003; Table 1, site 8)
O. latipes	Hd-rR	Hd-rR	B-II	B27	1	1	Lab stock
O. latipes	Himedaka	him	B-II, B-VII	-	4	4	Commercial fish
O. sakaizumii	HNI	HNI	A-I	A1	1	1	Lab stock
O. sakaizumii	Kinosaki	Kin	A-III	A14	4	3	Wild collection (Takehana et al. 2003; Table 1, site 178)
O. sakaizumii	Nanao	Nan	A-II	A11(3), A9*(2)	4	2	Wild collection (Takehana et al. 2003; Table 1, site 111)
O. sakaizumii	Higashidori	Hig	A-I	A1(1), A13*(3)	4	2	Wild collection (Takehana et al. 2003; Table 1, site 2)

*1: mtDNA subclade defined by the partial sequence of *cyt* b (1241 bp) obtained by Sanger sequencing and analyzed by the neighbor-joining (NJ) algorithm; *2: Mitotype defined by the partial sequence of the mitochondrial *cytochrome b* gene (1241 bp) obtained by PCR-RFLP analysis. *3: Due to the small number of sequenced reads, some individuals were excluded from the population structure analysis.

Table S2. Species list used for calculating the genome-wide kMER frequencies

Phylum	Species	Accession ID	Contig	Total bases
Porifera	*Amphimedon queenslandica*	GCA_000090795.1	13397	166679601
Cnidaria	*Nematostella vectensis*	GCA_000209225.1	10804	356613585
Ctenophora	*Mnemiopsis leidyi*	GCA_000226015.1	5101	155875873
Platyhelminthes	*Schistosoma mansoni*	GCA_000237925.2	3	127906668
Nematoda	*Caenorhabditis elegans*	GCA_000002985.3	2	30351855
Nematoda	*Loa loa*	GCA_000183805.3	5773	91373458
Arthropoda	*Aedes aegypti*	GCA_000004015.3	4757	1383974186
Arthropoda	*Zootermopsis nevadensis*	GCA_000696155.1	31662	485009472
Arthropoda	*Daphnia magna*	GCA_001632505.1	28801	129543483
Annelida	*Capitella teleta*	GCA_000328365.1	20803	333283208
Annelida	*Helobdella robusta*	GCA_000327385.1	1991	235376169
Mollusca	*Crassostrea gigas*	GCA_000297895.1	7658	557717710
Mollusca	*Octopus bimaculoides*	GCA_001194135.1	151674	2338188782
Brachiopoda	*Lingula anatina*	GCA_001039355.1	3830	425494968
Echinodermata	*Strongylocentrotus purpuratus*	GCA_000002235.2	32008	936564995
Vertebrata	*Oryzias latipes*	GCA_002234675.1	24	734040372
Vertebrata	*Homo sapiens*	GCA_000001405.15	23	3088269832

Table ~~S2~~S3. List of PCR primers examined by electrophoresis or by sequencing

Primer name	Forward primers (adapter + spacer + target)	Reverse primers (adapter + spacer + target)	Miseq
10mer-L1	CGCTCTTCCGATCTNNNNNNNAAATAAAATG	TGCTCTTCCGATCTNNNNNNNAAATAAAATG	
10mer-L2	CGCTCTTCCGATCTNNNNNNNAATTCAAAAT	TGCTCTTCCGATCTNNNNNNNAATTCAAAAT	
10mer-L3	CGCTCTTCCGATCTNNNNNNNTTGTTTTGTT	TGCTCTTCCGATCTNNNNNNNTTGTTTTGTT	
10mer-L4	CGCTCTTCCGATCTNNNNNNNATTTGTTTTT	TGCTCTTCCGATCTNNNNNNNATTTGTTTTT	
10mer-L5	CGCTCTTCCGATCTNNNNNNNATTTTCATTT	TGCTCTTCCGATCTNNNNNNNATTTTCATTT	
10mer-L6	CGCTCTTCCGATCTNNNNNNNCATTTTGTTT	TGCTCTTCCGATCTNNNNNNNCATTTTGTTT	+
10mer-L7	CGCTCTTCCGATCTNNNNNNNTATTTGTTTT	TGCTCTTCCGATCTNNNNNNNTATTTGTTTT	
10mer-L8	CGCTCTTCCGATCTNNNNNNNTATTTTTCTT	TGCTCTTCCGATCTNNNNNNNTATTTTTCTT	
10mer-L9	CGCTCTTCCGATCTNNNNNNNTTCATTTTTG	TGCTCTTCCGATCTNNNNNNNTTCATTTTTG	
10mer-L10	CGCTCTTCCGATCTNNNNNNNTTCTTTGTTT	TGCTCTTCCGATCTNNNNNNNTTCTTTGTTT	
10mer-L11	CGCTCTTCCGATCTNNNNNNNTTGTTTTTGT	TGCTCTTCCGATCTNNNNNNNTTGTTTTTGT	
10mer-L12	CGCTCTTCCGATCTNNNNNNNTTTATGTTTT	TGCTCTTCCGATCTNNNNNNNTTTATGTTTT	
10mer-L13	CGCTCTTCCGATCTNNNNNNNTTTCATTTTG	TGCTCTTCCGATCTNNNNNNNTTTCATTTTG	
10mer-L14	CGCTCTTCCGATCTNNNNNNNTTTCTTGTTT	TGCTCTTCCGATCTNNNNNNNTTTCTTGTTT	+
10mer-L15	CGCTCTTCCGATCTNNNNNNNTTTGTTTCTT	TGCTCTTCCGATCTNNNNNNNTTTGTTTCTT	
10mer-L16	CGCTCTTCCGATCTNNNNNNNTTTTTGTATT	TGCTCTTCCGATCTNNNNNNNTTTTTGTATT	
10mer-M1	CGCTCTTCCGATCTNNNNNNNAATTTGTCGT	TGCTCTTCCGATCTNNNNNNNAATTTGTCGT	+
10mer-M2	CGCTCTTCCGATCTNNNNNNNCTTGATTCGT	TGCTCTTCCGATCTNNNNNNNCTTGATTCGT	
10mer-M3	CGCTCTTCCGATCTNNNNNNNGTATCGTATT	TGCTCTTCCGATCTNNNNNNNGTATCGTATT	
10mer-M4	CGCTCTTCCGATCTNNNNNNNTGATCGGTTT	TGCTCTTCCGATCTNNNNNNNTGATCGGTTT	
10mer-S1	CGCTCTTCCGATCTNNNNNNNCCCGGTCGTG	TGCTCTTCCGATCTNNNNNNNCCCGGTCGTG	
10mer-S2	CGCTCTTCCGATCTNNNNNNNCGTACCCCGT	TGCTCTTCCGATCTNNNNNNNCGTACCCCGT	
10mer-S3	CGCTCTTCCGATCTNNNNNNNCGTACCCGCC	TGCTCTTCCGATCTNNNNNNNCGTACCCGCC	
10mer-S4	CGCTCTTCCGATCTNNNNNNNCGCCGTACCC	TGCTCTTCCGATCTNNNNNNNCGCCGTACCC	+
7mer-L1	CGCTCTTCCGATCTNNNNNNNAATTTTC	TGCTCTTCCGATCTNNNNNNNAATTTTC	
7mer-L2	CGCTCTTCCGATCTNNNNNNNATTTGTT	TGCTCTTCCGATCTNNNNNNNATTTGTT	
7mer-L3	CGCTCTTCCGATCTNNNNNNNATTTTCA	TGCTCTTCCGATCTNNNNNNNATTTTCA	
7mer-L4	CGCTCTTCCGATCTNNNNNNNATTTTTC	TGCTCTTCCGATCTNNNNNNNATTTTTC	
7mer-L5	CGCTCTTCCGATCTNNNNNNNCATTTTG	TGCTCTTCCGATCTNNNNNNNCATTTTG	
7mer-L6	CGCTCTTCCGATCTNNNNNNNGTTTTGT	TGCTCTTCCGATCTNNNNNNNGTTTTGT	
7mer-L7	CGCTCTTCCGATCTNNNNNNNTATTTGT	TGCTCTTCCGATCTNNNNNNNTATTTGT	

7mer-L8	CGCTCTTCCGATCTNNNNNNNTATTTTT	TGCTCTTCCGATCTNNNNNNNTATTTTT	
7mer-L9	CGCTCTTCCGATCTNNNNNNNTGTTTTC	TGCTCTTCCGATCTNNNNNNNTGTTTTC	
7mer-L10	CGCTCTTCCGATCTNNNNNNNTTCATTT	TGCTCTTCCGATCTNNNNNNNTTCATTT	
7mer-L11	CGCTCTTCCGATCTNNNNNNNTTCTTTG	TGCTCTTCCGATCTNNNNNNNTTCTTTG	
7mer-L12	CGCTCTTCCGATCTNNNNNNNTTTATGT	TGCTCTTCCGATCTNNNNNNNTTTATGT	+
7mer-L13	CGCTCTTCCGATCTNNNNNNNTTTCTTG	TGCTCTTCCGATCTNNNNNNNTTTCTTG	
7mer-L14	CGCTCTTCCGATCTNNNNNNNTTGTTTT	TGCTCTTCCGATCTNNNNNNNTTGTTTT	
7mer-L15	CGCTCTTCCGATCTNNNNNNNTTTTTGT	TGCTCTTCCGATCTNNNNNNNTTTTTGT	+
7mer-L16	CGCTCTTCCGATCTNNNNNNNTTTTATG	TGCTCTTCCGATCTNNNNNNNTTTTATG	
7mer-M1	CGCTCTTCCGATCTNNNNNNNAAAATTC	TGCTCTTCCGATCTNNNNNNNAAAATTC	
7mer-M2	CGCTCTTCCGATCTNNNNNNNAATTTGT	TGCTCTTCCGATCTNNNNNNNAATTTGT	
7mer-M3	CGCTCTTCCGATCTNNNNNNNTTTGATC	TGCTCTTCCGATCTNNNNNNNTTTGATC	+
7mer-M4	CGCTCTTCCGATCTNNNNNNNGTATCGT	TGCTCTTCCGATCTNNNNNNNGTATCGT	
7mer-S1	CGCTCTTCCGATCTNNNNNNNACCCGTC	TGCTCTTCCGATCTNNNNNNNACCCGTC	
7mer-S2	CGCTCTTCCGATCTNNNNNNNCGGACTC	TGCTCTTCCGATCTNNNNNNNCGGACTC	
7mer-S3	CGCTCTTCCGATCTNNNNNNNGTCGCCC	TGCTCTTCCGATCTNNNNNNNGTCGCCC	+
7mer-S4	CGCTCTTCCGATCTNNNNNNNTCGGGTC	TGCTCTTCCGATCTNNNNNNNTCGGGTC	

Table ~~S2~~S3. Primer sequences used for the MIG-seq cocktail*

Name	Sequences (5′–3′)
Forward primers	
(ACT)4TG-f	CGCTCTTCCGATCTCTGACTACTACTACTTG
(CTA)4TG-f	CGCTCTTCCGATCTCTGCTACTACTACTATG
(TTG)4AC-f	CGCTCTTCCGATCTCTGTTGTTGTTGTTGAC
(GTT)4CC-f	CGCTCTTCCGATCTCTGGTTGTTGTTGTTCC
(GTT)4TC-f	CGCTCTTCCGATCTCTGGTTGTTGTTGTTTC
(GTG)4AC-f	CGCTCTTCCGATCTCTGGTGGTGGTGGTGAC
(GT)6TC-f	CGCTCTTCCGATCTCTGGTGTGTGTGTGTTC
(TG)6AC-f	CGCTCTTCCGATCTCTGTGTGTGTGTGTGAC
(ACT)4TG-f	CGCTCTTCCGATCTCTGACTACTACTACTTG
Reverse primers	
(ACT)4TG-r	TGCTCTTCCGATCTGACACTACTACTACTTG
(CTA)4TG-r	TGCTCTTCCGATCTGACCTACTACTACTATG
(TTG)4AC-r	TGCTCTTCCGATCTGACTTGTTGTTGTTGAC
(GTT)4CC-r	TGCTCTTCCGATCTGACGTTGTTGTTGTTCC
(GTT)4TC-r	TGCTCTTCCGATCTGACGTTGTTGTTGTTTC
(GTG)4AC-r	TGCTCTTCCGATCTGACGTGGTGGTGGTGAC
(GT)6TC-r	TGCTCTTCCGATCTGACGTGTGTGTGTGTTC
(TG)6AC-r	TGCTCTTCCGATCTGACTGTGTGTGTGTGAC

Reference

Suyama, Y., & Matsuki, Y. (2015). MIG-seq : an effective PCR- based method for genome-wide single-nucleotide polymorphism genotyping using the next-generation sequencing platform. *Scientific Reports*, 5(16963). doi: 10.1038/srep16963

Table S3. Average individual throughput with and without the reference genome

Pipeline	Fastq	Preprocess		Mapping		Genotyping
	Read number (A) ± SE	Read number (B) ± SE	B/A	Mapped read number (C) ± SE	C/A	DP≥1 ± SE
BWA and GATK	2342028 ± 277218	2154350 ± 242875	0.93	1494969 ± 175777	0.64	16662303 ± 825481
Stacks (M = 7)		1364654 ± 155865	0.59	757607 ± 90716	0.29	5058211 ± 472091

Table S4. Variable sites in the population structure dataset

		Variable sites (SNVs + InDels)		
Pipeline	Genotyping rate	Total bases	*O. latipes* (*N* = 59)	*O. sakaizumii* (*N = 8)*
BWA and GATK	≥ 0.6	80665	57386	13192
	≥ 0.8	46741	31859	8698
	= 1.0	6479	4617	1265
Stacks	≥ 0.6	42134	30895	7480
	≥ 0.8	18594	13593	3071
	= 1.0	1362	1006	213

Table S5. Genome-wide average nucleotide diversity (*Pi*), pairwise genetic distances D_{XY} based on the total sequences (126765 bp with 6479 SNVs).

Pi	Hig	Nan	Kin	Ich	Iso	Tsu	Mis	Kum	Hik	Hof	Miy	Sus	Yam	Nak	Kun
	0.0004	0.0008	0.0014	0.0014	0.0012	0.0014	0.0011	0.0009	0.0012	0.0025	0.0024	0.0011	0.0029	0.0015	0.0006

Dxy	Hig	Nan	Kin	Ich	Iso	Tsu	Mis	Kum	Hik	Hof	Miy	Sus	Yam	Nak	Kun
Hig															
Nan	0.0014														
Kin	0.0034	0.0034													
Ich	0.0089	0.0089	0.0088												
Iso	0.0092	0.0092	0.0090	0.0022											
Tsu	0.0092	0.0092	0.0090	0.0023	0.0017										
Mis	0.0092	0.0092	0.0090	0.0024	0.0020	0.0019									
Kum	0.0091	0.0091	0.0089	0.0027	0.0024	0.0023	0.0024								
Hik	0.0093	0.0094	0.0092	0.0032	0.0031	0.0031	0.0031	0.0030							
Hof	0.0094	0.0094	0.0093	0.0036	0.0035	0.0035	0.0034	0.0034	0.0035						
Miy	0.0095	0.0095	0.0093	0.0037	0.0036	0.0036	0.0035	0.0034	0.0035	0.0034					
Sus	0.0100	0.0100	0.0098	0.0043	0.0042	0.0043	0.0042	0.0042	0.0043	0.0043	0.0044				
Yam	0.0099	0.0099	0.0098	0.0048	0.0048	0.0048	0.0048	0.0047	0.0049	0.0047	0.0049	0.0052			
Nak	0.0097	0.0097	0.0095	0.0046	0.0045	0.0045	0.0045	0.0044	0.0046	0.0046	0.0045	0.0050	0.0041		
Kun	0.0097	0.0097	0.0095	0.0048	0.0047	0.0048	0.0048	0.0047	0.0048	0.0049	0.0049	0.0052	0.0042	0.0025	

Table S6. Genome-wide weighted *Fst* based on the 6479 SNVs.

Fst	Hig	Nan	Kin	Ich	Iso	Tsu	Mis	Kum	Hik	Hof	Miy	Sus	Yam	Nak	Kun
Hig		0.52	0.72	0.88	0.89	0.87	0.90	0.91	0.89	0.79	0.79	0.91	0.78	0.88	0.94
Nan			0.67	0.86	0.88	0.86	0.89	0.90	0.88	0.77	0.78	0.90	0.77	0.87	0.92
Kin				0.84	0.86	0.84	0.86	0.87	0.86	0.77	0.77	0.88	0.76	0.85	0.89
Ich					0.43	0.38	0.50	0.59	0.60	0.46	0.48	0.73	0.55	0.70	0.78
Iso						0.23	0.43	0.57	0.61	0.46	0.49	0.73	0.58	0.70	0.79
Tsu							0.34	0.50	0.56	0.43	0.46	0.70	0.55	0.68	0.76
Mis								0.58	0.62	0.47	0.49	0.74	0.59	0.72	0.80
Kum									0.65	0.49	0.51	0.77	0.61	0.73	0.83
Hik										0.45	0.45	0.74	0.57	0.71	0.80
Hof											0.27	0.57	0.42	0.57	0.64
Miy												0.58	0.45	0.56	0.64
Sus													0.62	0.75	0.83
Yam														0.47	0.54
Nak															0.55
Kun															

Table S7. Cluster number (K) and cross validation errors in ADMIXTURE analysis

K	CV error
1	0.4899
2	0.3828
3	0.3072
4	0.2785
5	0.2601
6	0.2452
7	0.2330
8	0.2253
9	0.2182
10	0.2143
11	0.2098
12	0.2072
13	0.2087
14	0.2132
15	0.2203

Table S8. Inferred source population parameters for two-way admixtures of the Himedaka strain using MixMapper

Admixed	Source 1	Source 2	Replicates	Residual norm	α	Branch 1	Branch 2	Mixed drift
Himedaka	Hikone	Mishima	66	2.56E-06	0.419-0.664	0.010-0.019 / 0.033	0.000-0.005 / 0.016	0.004-0.007
	(Isobe, Mishima and Tsu)	Hikone	25	2.73E-06	0.447-0.708	0.001-0.006 / 0.006	0.010-0.028 / 0.032	0.004-0.008
	(Isobe and Tsu)	Hikone	9	2.15E-06	0.401-0.598	0.000-0.002 / 0.007	0.012-0.022 / 0.036	0.005-0.008

Parameters for an admixture scenario calculated based on 100 bootstrap replicates: Source 1, and Source 2 are the inferred source populations; α is the 95% bootstrap confidence interval for the ancestral proportion from Source 1; Branch1 and Branch2 are the drift distances for each source branch (estimated range of drift before admixture / total drift of source branch); and Mixed drift represents drift following admixture.

Table S9. Results of Patterson’s D statistic to test admixture between *O. latipes* populations in eastern Honshu and *O. sakaizumii*

W	X	Y	Z	*D*	SE	*Z*	BABA	ABBA
Mishima	Ichinoseki	Kunigami	*O. sakaizumii*	0.27	0.15	1.81	21	12
Mishima	Isobe	Kunigami	*O. sakaizumii*	-0.01	0.26	-0.03	9	9
Mishima	Tsu	Kunigami	*O. sakaizumii*	0	0.25	0.01	8	8
Ichinoseki	Mishima	Kunigami	*O. sakaizumii*	-0.27	0.15	-1.81	12	21
Ichinoseki	Isobe	Kunigami	*O. sakaizumii*	-0.32	0.16	-1.95	10	18
Ichinoseki	Tsu	Kunigami	*O. sakaizumii*	-0.31	0.16	-1.97	10	19
Isobe	Mishima	Kunigami	*O. sakaizumii*	0.01	0.26	0.03	9	9
Isobe	Ichinoseki	Kunigami	*O. sakaizumii*	0.32	0.16	1.95	18	10
Isobe	Tsu	Kunigami	*O. sakaizumii*	0	0.19	0.02	5	5
Tsu	Mishima	Kunigami	*O. sakaizumii*	0.00	0.25	-0.01	8	8
Tsu	Ichinoseki	Kunigami	*O. sakaizumii*	0.31	0.16	1.97	19	10
Tsu	Isobe	Kunigami	*O. sakaizumii*	0.00	0.19	-0.02	5	5

We include *O. sakaizumii* and *O. latipes* populations from the western Kyushu clade (Kunigami), and four populations collected from the eastern part of Honshu (Mishima, Tsu, Isobe, and Ichinoseki populations).

Table S10. Inferred source population parameters for two-way admixtures of the Ichinoseki population in MixMapper

Admixed	Source 1	Source 2	Replicates	Residual norm	α	Branch 1	Branch 2	Mixed drift
Ichinoseki	Isobe	(Kunigami, root and *O. sakaizumii*)	62	1.07E-05	0.433-0.759	0.001-0.006 / 0.009	0.014-0.024 / 0.025	0.017-0.023
	Isobe	Hikone	16	1.49E-05	0.491-0.717	0.002-0.005 / 0.009	0.000-0.006 / 0.034	0.017-0.022
	(Isobe and Tsu)	(root and *O. sakaizumii*)	7	2.30E-05	0.813-0.920	0.001-0.004 / 0.005	0.015-0.028 / 0.043	0.015-0.020
	Isobe	(Isobe, Kumano, Mishima and Tsu)	3	8.86E-06	0.253-0.562	0.002-0.009 / 0.009	0.003-0.008 / 0.013	0.015-0.018
	(Isobe and Tsu)	Hikone	3	2.13E-05	0.703-0.775	0.003-0.006 / 0.007	0.003-0.008 / 0.031	0.015-0.017
	(Isobe and Tsu)	(Kunigami, root and *O. sakaizumii*)	3	1.90E-05	0.624-0.749	0.002-0.003 / 0.006	0.014-0.020 / 0.026	0.018-0.019
	(Isobe, Mishima and Tsu)	*O. sakaizumii*	2	3.55E-05	0.912-0.939	0.004-0.005 / 0.006	0.012-0.014 / 0.096	0.018-0.022
	Isobe	(root and *O. sakaizumii*)	1	1.08E-05	0.801-n/a	0.001-n/a / 0.01	0.034-n/a / 0.042	0.020-n/a
	(Isobe, Mishima and Tsu)	(root and *O. sakaizumii*)	1	4.18E-05	0.883-n/a	0.007-n/a / 0.01	0.004-n/a / 0.043	0.019-n/a
	(Isobe and Tsu)	*O. sakaizumii*	1	1.53E-05	0.909-n/a	0.002-n/a / 0.006	0.014-n/a / 0.092	0.016-n/a
	Tsu	(Kunigami, root and *O. sakaizumii*)	1	2.04E-05	0.665-n/a	0.001-n/a / 0.005	0.022-n/a / 0.026	0.017-n/a

Parameters for an admixture scenario calculated based on 100 bootstrap replicates: Source 1, and Source 2 are the inferred source populations; α is the 95% bootstrap confidence interval for the ancestral proportion from Source 1; Branch 1 and Branch 2 are the drift distances for each source branch (estimated range of drift before admixture / total drift of source branch); and Mixed drift represents drift following admixture.

Table S1. Collection site, mitochondrial DNA genotype and number of samples

Species	Collection site / Strain	Abbreviation	mtDNA subclade *1	Mitotype *2	Number genotyped	Number analyzed *3	Source of sample
O. latipes	Kunigami	Kunigami	B-XI	B24*(2)	3	3	Wild collection (This study)
O. latipes	Yamato	Yam	B-XI	B24*(5)	5	4	Wild collection (Takehana et al. 2003; Table 1, site 258)
O. latipes	Nakatane	Nak	B-VIII	B8*(5)	5	5	Wild collection (Takehana et al. 2003; Table 1, site 293)
O. latipes	Miyazaki	Miy	B-VIII	B4*(5)	5	5	Wild collection (Takehana et al. 2003; Table 1, site 279)
O. latipes	Hofu	Hof	B	B13*(5)	5	5	Wild collection (Takehana et al. 2003; Table 1, site 227)
O. latipes	Susaki	Sus	B-V	B1d*(5)	4	4	Wild collection (Takehana et al. 2003; Table 1, site 245)
O. latipes	Hikone	Hik	B-VII	B22*(5)	4	4	Wild collection (Takehana et al. 2003; Table 1, site 158)
O. latipes	Kumano	Kum	B-VI	B33*(5)	5	5	Wild collection (Takehana et al. 2003; Table 1, site 155)
O. latipes	Tsu	Tsu	B-II	B1c(5)	5	5	Wild collection (Takehana et al. 2003; Table 1, site 147)
O. latipes	Isobe	Iso	B	B1c(1), B5(3), B11(1)	5	5	Wild collection (Takehana et al. 2003; Table 1, site 152)
O. latipes	Mishima	Mis	B-IV	B18*(5)	5	5	Wild collection (Takehana et al. 2003; Table 1, site 136)
O. latipes	Ichinoseki	Ich	B	B11(5)	5	4	Wild collection (Takehana et al. 2003; Table 1, site 8)
O. latipes	Hd-rR	Hd-rR	B-II	B27	1	1	Lab stock
O. latipes	Himedaka	him	B-II, B-VII	-	4	4	Commercial fish
O. sakaizumii	HNI	HNI	A-I	A1	1	1	Lab stock
O. sakaizumii	Kinosaki	Kin	A-III	A14	4	3	Wild collection (Takehana et al. 2003; Table 1, site 178)
O. sakaizumii	Nanao	Nan	A-II	A11(3), A9*(2)	4	2	Wild collection (Takehana et al. 2003; Table 1, site 111)
O. sakaizumii	Higashidori	Hig	A-I	A1(1), A13*(3)	4	2	Wild collection (Takehana et al. 2003; Table 1, site 2)

*1: mtDNA subclade defined by the partial sequence of *cyt* b (1241 bp) obtained by Sanger sequencing and analyzed by the neighbor-joining (NJ) algorithm; *2: Mitotype defined by the partial sequence of the mitochondrial *cytochrome b* gene (1241 bp) obtained by PCR-RFLP analysis. *3: Due to the small number of sequenced reads, some individuals were excluded from the population structure analysis.

97
